# Early renal damage among children living in the region of highest burden of chronic kidney disease of unknown etiology (CKDu) in Sri Lanka

**DOI:** 10.1186/s12882-018-0911-8

**Published:** 2018-05-16

**Authors:** S. B. Agampodi, G. S. Amarasinghe, P. G. C. R. Naotunna, C. S. Jayasumana, S. H. Siribaddana

**Affiliations:** 1 Department of Community Medicine, Faculty of Medicine and Allied Sciences, Rajarata University of Sri Lanka, Saliyapura, Sri Lanka; 2 Department of Pharmacology, Faculty of Medicine and Allied Sciences, Rajarata University of Sri Lanka, Saliyapura, Sri Lanka; 3 Department of Medicine, Faculty of Medicine and Allied Sciences, Rajarata University of Sri Lanka, Saliyapura, Sri Lanka

## Abstract

**Background:**

Chronic kidney disease of unknown origin (CKDu) in Sri Lanka is grouped with several other epidemics of similar nature across the world as Chronic Interstitial Nephritis in Agricultural Communities (CINAC). In CKDu endemic countries, the focus has mainly been on adults. We hypothesized that studying distribution and factors associated with elevated urine albumin to creatinine ratio (UACR), an early marker of kidney injury, among children living in a CKDu endemic area may provide important clues about the onset and progression of the disease.

**Methods:**

This cross sectional study was performed in rural primary schools in North Central Province of Sri Lnaka, a CKDu high endemic region. Total of 2880 students aging 5 to 11 years from 67 schools were enrolled for urinalysis in a random spot urine sample. Bedside Schwartz formula was used to measure estimated glomerular filtration rate (eGFR) on all children with UACR > 30 mg/g in Polonnaruwa district and a group of age matched controls. A standard multiple linear regression using log transformed UACR as the dependent variable was performed. Mean eGFR were compared between UACR elevated group and controls using independent sample t test.

**Results:**

Median UACR was 10.3 mg/g. Sex, ethnicity, history of having a chronic disease and age uniquely contributed to the multiple regression model which only explained 2.8% of the variance in the log of the UACR (*p* < 0.001). Only 15 (0.5%) had UACR> 300 mg/g while 8.2% (*n* = 236) had UACR between 30 to 300 mg/g and 89.8% (*n* = 203) of them did not have a chronic disease (Chi square 2.21, *p* = 0.091). Mean eGFR was significantly lower in the group with elevated UACR (88.9 mg/dl/1.73 m2, 95% CI for mean 86.4- 91.3) compared to group with normal UACR (93.7 mg/dl/1.73 m2,95% CI 91.1- 96.3) (t 2.7, p 0.007). Three out of the four students with eGFR less than 60 mg/dl/1.73 m2 had moderately elevated UACR.

**Conclusion:**

This study provides evidence to suggest that children in CKDu endemic regions are having an early renal damage. This observation needs to be investigated further in order to understand the worldwide epidemic of CKDu.

## Background

Epidemics of chronic kidney disease of unknown origin (CKDu) resulting in progressive deterioration of renal function causing end stage renal failure that cannot be linked to a known causative factor are reported from different regions of the world including areas of Sri Lanka, India and Central America [1–3]. CKDu in Sri Lanka and Mesoamerican nephropathy demonstrate clinicopathological similarities and are also referred to as Chronic Interstitial Nephritis in Agricultural Communities (CINAC) [4]. Though exact etiology has not been confirmed yet, multiple factors such as exposure to environmental toxins, heavy metals, heat stress, and genetic susceptibility are assumed to be responsible for origin of the disease [5].

Renal insult leading to CKDu may start on a very early age, specially due to exposure to environmental toxins [6]. However, in countries where CKDu is a major public health issue, the focus is mainly on adult age groups. In Sri Lanka, a country with a heavy burden of CKDu, neither the screening programmes nor the studies pay attention on children and possibility of early renal damage. Nevertheless, young patients have been reported in some studies from Sri Lanka. For an example, 2 (7.7%) out of 26 less than 20 years old individuals participating in a screening programme in Madawachiya (Anuradhapura district, Sri Lanka) were reported to have stage 3-5 CKD (GFR less than 60 ml/min/1.73 m^2^) [7]. According to a hospital based study, 11.2% CKDu patients treated at the tertiary care center of Anuradhapura district were between 15 to 30 years of age [8]. CKDu has been reported in 10-15 years old children in North Central Province (NCP) as well [9]. Significance of these findings on describing epidemiology of CKDu in young population is limited by small number of younger participants and need for different Glomerular filtration rate (GFR) estimation methods for children.

Screening children for renal problems is predominantly done using urinalysis due to none-invasive nature of the test. It has been used in several countries across the world with varying success in identifying and preventing permanent renal diseases [10]. School based urinalysis programmes, specially in Asian countries such as Japan and Korea have shown success in reducing the progress to end stage renal disease through early identification of asymptomatic renal diseases [11, 12].

Urine albumin to creatinine ratio (UACR) in a spot sample is an early marker of kidney injury [13, 14]. Further, the presence of proteinuria has also been described as a risk factor for rapid progression and poorer prognosis of CKD [15, 16]. We hypothesized that studying distribution and factors associated with elevated UACR among children living in a CKDu endemic area may provide important clues about the onset and progression of the disease. We report here the first Sri Lankan study on renal functions of children in the province with the highest burden of CKDu.

## Methods

### Study design

This is a part of a large school based cross sectional study conducted among rural primary school children in North Central Province (NCP). Detailed methodology of the larger study has been described elsewhere [17].

### Study setting

The study was carried out in Anuradhapura and Polonnaruwa districts of the NCP, in the dry zone of Sri Lanka. Estimated population of Anuradhapura and Polonnaruwa districts were 893,000 and 419,000 respectively in 2015 [18]. Majority of the population engage in paddy cultivation [19]. According to ministry of health, approximately 1000 and 500 CKD/CKDu patients has been reported annually from Anuradhapura and Polonnaruwa districts between 2011 to 2015 [20].

Data collection was carried out in October to December 2014.

### Study population

School children studying in grade 1 to 5 (aging 5 to 11 years) attending very difficult and difficult schools (classified according to number of students, accessibility and facilities available at the school) in NCP were the study population for the present study.

### Study sample

The sampling frame for the original study included 701 rural primary schools in the province and 100 schools were selected using a multi stage cluster sampling. From each school, 50 students in 5 to 11 years age group were enrolled randomly.

From the original sample, 2880 children from 67 schools from five educational zones representing both Anuradhapura and Polonnaruwa districts were selected for the present study. From this, children attending 42 schools from Polonnaruwa district were selected for eGFR analysis. It included all the children with elevated UACR (> 30 mg/g) in Polonnaruwa and an age matched sample of children with UACR less than 30 mg/g.

### Variables

We used district, sex, age, ethnicity, family history of kidney disease, history of having a chronic disease, drinking water source, BMI for age (z value) and hemoglobin concentration as predictor variables.

Several methods are available to estimate urine albumin excretion. Urine albumin excretion in a 24-h urine collection is considered the best bust is difficult to be assessed specially in children. Albumin and albumin to creatinine ratio in a random spot urine sample are more convenient methods that are used [21].

National Kidney Foundation (NKF) reference ranges for UACR was used to classify urine albumin excretion (UACR< 30 mg/g – normal, 30-300 mg/g –moderately increased, > 300 mg/g – severely increased [21].

Serum creatinine (SCr) was assessed using IDMS traceable modified Jaffe method. GFR was calculated using the beside Schwartz formula (eGFR = kL/SCr, k = 0.413). Though early stages of CKD may have GFR levels below 90 mg/dl/1.73m^2^, GFR < 60 mg/dl/1.73 m^2^ was used as the cutoff for low GFR [16].

The WHO cutoff upper limit of the reference range (11.5 g/dl) was used to define anemia [22].

### Data sources/measurement

A self-administered questionnaire was sent to parents prior to the day of data collection. Trained medical graduates measured weight and height. A random spot urine sample was collected on the day of data collection. Trained nurses drew blood samples. Investigation reports were obtained from a commercial diagnostic laboratory with external quality control methods.

### Data analysis

Urine albumin and UACR showed skewed distribution hence we used Mann-Whitney U test and Kruskel Wallis test. Since data was skewed we performed a standard multiple linear regression using log transformed UACR as the dependent variable. District (Anuradhapura and Polonnaruwa), Sex (Male and Female), Ethnicity (Sinhalese or other), Family history of CKD (No, Yes), Drinking water source (from approved wells or filtered or bottled water or springs or from other sources including unapproved wells, water supply schemes, ponds, and tube wells), Hemoglobin (g/dl), BMI for age (z score) and age (in months) were entered as dependent variables. Beta coefficients were exponentiated to interpret the results. Correlation was tested between BMI for age (z value) and log transformed urine albumin and UACR.

We compared the mean eGFR value of UACR elevated group in Polonnaruwa with that of age matched controls with normal UACR using independent sample t test.

SPSS version 20 was used for data analysis. Diagrams were created using GraphPad Prism.

## Results

Altogether, 2880 school children aging 5-10 years were included in the sample. Male to female ratio was almost 1:1. Majority (86.7%) were Sinhalese. Of 2880 children studied, 580 reported CKD among their parents/ grandparents (Table 1).

**Table 1:** Characteristics of the 2880 rural primary school children from North Central Province, Sri Lanka

	N	%
District		
Anuradhapura	1014	35.2
Polonnaruwa	1866	64.8
Education Division		
Anuradhapura	364	12.6
Dibulagala	517	18.0
Higurakgoda	818	28.4
Kabathigollawa	650	22.6
Polonnaruwa	531	18.4
Age category		
5 years	216	7.5
6 years	677	23.5
7 years	598	20.8
8 years	606	21.0
9 years	574	19.9
10 years	209	7.3
Sex		
Female	1412	49.0
Male	1453	50.5
Ethnicity		
Sinhalese	2298	86.7
Moor/Malay	347	13.1
Tamil	4	0.2
History of any chronic disease		
Yes	293	7.5
No	3597	92.5
Family history of CKD		
No	3353	85.3
Yes	580	14.7

**Fig. 1:**
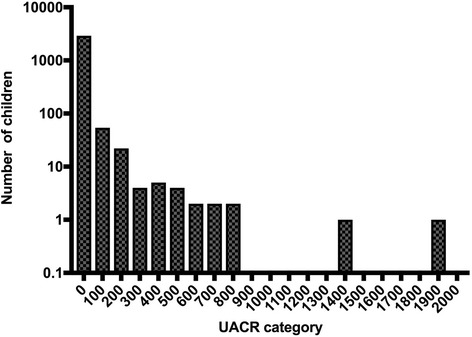
Distribution of UACR among 2880 rural primary school children from North Central Province, Sri Lanka

Distributions of urine albumin, urine creatinine and UACR (Fig. 1) were skewed. The median values for urine albumin, creatinine and UACR were 7 mg (range 2-902), 71.5 g (range 9-289) and 12.6 mg/g (range 1-784) respectively (Table 2).

**Table 2:** Distribution of urine albumin, urine creatinine, and urine albumin to creatinine ratio (UACR) among 2880 rural primary school children from North Central Province, Sri Lanka

	Median	Mean (95% CI)	SD	Maximum	Minimum
Urine albumin (mg)	6	12.4 (10.8-13.9)	42.8	902	2
Urine creatinine (g)	67	74.1(72.6-75.7)	41.3	289	9
UACR (mg/g)	10.3	18.3(16.7-19.9)	44.0	784	1

District, sex, ethnicity and having a history of chronic disease showed statistically significant difference in the mean ranks of UACR between the groups but the effect sizes were small in all of them [23] (See Table 3).

**Table 3:** Comparison of UACR (mg/g) between groups using Mann-Whitney U test for binary independent variables

		Median	Z	*p*	Approximate effect size
District	Anuradhapura	10.74	−2.217	0.027	−0.04
	Polonnaruwa	10.00			
Sex	Males	9.43	−6.633	0.001	−0.12
	Females	11.29			
Ethnicity	Sinhala	10.64	−4.357	0.001	−0.09
	Other	9.09			
Family History of CKD	No	10.25	−0.833	0.405	−0.02
	Yes	10.53			
Chronic diseases	Yes	11.11	−2.047	0.041	−0.04
	No	10.38			
Anemia	No	10.11	−1.908	0.056	−0.04
	Yes	11.04			

Kruskal-Wallis test was performed to compare the median UACR values between categorical variables. None of the variables that were tested; age (in years) (Chi Square 10.787, p 0.056) drinking water source (Chi-Square 5.57, p 0.59), thinness (Chi-Square 7.0, p 0.14) wasting (Chi Square 2.38, p 0.31) and stunting (Chi-Square 0.69, p 0.71) revealed statistically significant difference in UACR between groups. There was no statistically significant correlation between the BMI for age and log converted urine albumin (Pearson r − 0.03, p 0.10) or log converted UACR (Pearson r − 0.03, p 0.13).

Standard multiple linear regression using log transformed UACR as the dependent variable showed that the model explain only 2.8% of the variance in the log of the UACR (*p* < 0.001). Sex, ethnicity, history of having a chronic disease and age make a unique contribution to the model. The exponentiated β values (done separately in Microsoft excel) showed that when all other variables stay the same, female sex leads to 1.14% increase in UACR while Sinhalese ethnicity leads to 0.95% increase in UACR and having a chronic disease leads to a 0.96% increase in UACR. For a month increase in age, UACR decreases by 0.96%. (Table 4).

**Table 4:** Results of the standard multiple linear regression using log UACR as the independent variable

Variable	N	exponentiated β	*p*
District (Anuradhapura)	2880	0.97	0.094
Sex (Male)	2865	1.14	0.000
Ethnicity (Sinhala)	2649	0.94	0.001
Family history of CKD (No)	2534	1.01	0.726
Drinking water source	2600	1.00	0.860
Hemoglobin (g/dl)	2880	1.01	0.787
BMI for age (z score)	2802	0.97	0.131
Age (in months)	2880	0.96	0.026
History of chronic disease (Yes)	2523	0.96	0.048

*N* number available for analysis, *β* Standardized Coefficient, *p* Significance of standardized coefficient

UACR was less than 30 mg/g among 2623 (91.3%) students while 8.2% (*n* = 236) had moderately elevated UACR. Only 15 (0.5%) had highly elevated UACR. Out of students with UACR more than 30 mg/g, 89.8% (*n* = 203) did not have a history of a chronic disease (Chi square 2.21, *p* = 0.091).

**Fig. 2:**
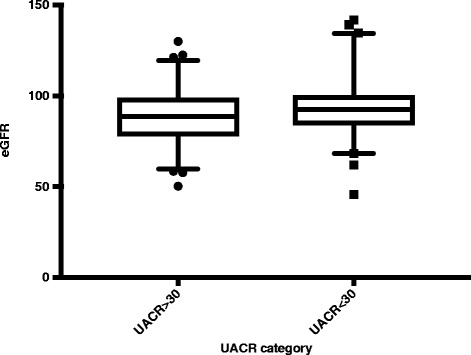
Distribution of eGFR among UACR normal and elevated groups of rural primary school children in Polonnaruwa

In the Polonnaruwa district, serum creatinine was done and eGFR was estimated for all children with elevated UACR (*n* = 141). Mean eGFR was 88.9 mg/dl/1.73 m^2^ (95% CI for mean 86.4- 91.3). Three (2.1%) had eGFR less than 60 mg/dl/1.73 m^2^ and another 74 (52.8%) were having eGFR 60-90 mg/dl/1.73 m^2^. All 3 students who had eGFR less than 60 mg/dl/1.73 m^2^ had moderately elevated UACR and only one of them had a history of chronic disease. To compare the eGFR among UACR elevated and normal groups we selected an age and district matched comparison group with normal ACR levels (*n* = 142). Mean eGFR in this sample was 93.7 mg/dl/1.73 m^2^ (95% CI 91.1- 96.3) and it was significantly higher than that of UACR elevated group (t 2.7, p 0.007). One of the children (0.7%) had eGFR less than 60 mg/dl/1.73 m^2^ and only 55 (44.4%) had eGFR within 60-90 mg/dl/1.73 m^2^ range (Fig. 2).

## Discussion

This is the largest study reported in scientific literature on renal functions of children living in CKDu endemic regions. In an area where CKDu is hyper endemic and considered the highest priority public health issue, we showed that 8.7% children are having elevated UACR. This seems to be higher compared to the prevalence reported in studies examining UACR in children of this age group from other countries. For an example prevalence of elevated UACR in 5-9 years old children from an Australian Aboriginal community where chronic kidney disease is highly prevalent was 5% [24]. Among 10 to 19 years old Korean children, microalbuminuria was reported as 3% [25]. According to a Japanese school based screening programme 2.5% had elevated UACR [26]. Another study in England reported that median UACR was 12.4, 5.9, 9.5, 8.0 mg/g among 6 to 11 years old white girls, white boys, black girls and black boys respectively [27]. Mean UACR was reported as 8.55 mg/g and 9.7 mg/g in 6 to 9 years old Spanish boys and girls respectively [28].

Albuminuria is a marker of different pathologies in both adults and children. Although upper limits for the reference range have not been specifically developed for children, it is considered similar to that of adults [21]. However, studies show that the association with adverse effects starts well below the recommended cutoff values [29, 30]. The normal level of albuminuria for young adults and children is considered to be 10 mg/g [16]. Microalbuminuria is associated with cardiovascular diseases and all-cause mortality in adults [16, 31] and metabolic syndrome and obesity in children [32]. Further, it is associated with renal injury in children due to variety of causes such as diabetes, hypertension, cardiovascular diseases, structural renal diseases, urinary tract infections and sickle cell disease [25, 32–37]. Urine albumin excretion is also proposed as a marker of generalized endothelial damage [37].

We observed that majority of children having elevated UACR were not having known chronic diseases; renal or other. This may be due to undetected renal injury or generalized endothelial damage from an unknown cause in these children. Urine albumin excretion can be increased in situations such as exercise, dehydration and orthostatic proteinuria [38]. To overcome the problem, an early morning sample is recommended to assess the albuminuria [39, 40]. In our study, we collected a random spot urine sample. However, the data collection was performed during morning hours of the day, usually before the recess when children are most likely to go and play out in the sun. Though using a random spot urine sample rather than a first void sample or repeated urine testing can overestimate the prevalence of elevated UACR [10] it is important to further investigate this finding to make sure that these children are not a target of CKDu prevailing in this area. Specially with our finding of lower mean eGFR levels among those who are having elevated UACR levels, the higher prevalence cannot be totally attributed to use of random urine sample.

We also observed that the mean GFR is lower even among the UACR normal group when compared to previous studies done in Iranian (99.66 ml/min/1.73 m^2^) [41] and Korean (99.24 ml/min/1.73m^2^) children [42]. Prevalence of low GFR (< 60 ml/min/1.73m^2^) was 1.7% among poor Mexican children under 18 years of age [43] and 1.26% (*N* = 9) among Iranian children [41]. Though there are difference in formula used and slight changes in the age groups, abnormalities we observed in UACR, mean eGFR and prevalence of low eGFR in this study sample poses an important question to answer; whether the CKDu in this area is having very early onset.

UACR vary with age, gender and race in children which was observed in this sample as well [32]. Epidemiological studies show that female sex, agricultural communities, family history, geographical clustering and consumption of surface water are associated with CKDu [5, 44]. We did not find that UACR in children was significantly affected by positive family history or water source.

As previously mentioned, interpretation of finding of this study should be done within the limitations of methodology. First, the urinalysis was done using a spot urine sample. We did not exclude children with acute or chronic renal diseases from the study sample. However, weather each child is having any chronic disease (including renal) was recorded. Since the data was collected from children attending the school on day of data collection, children suffering severe acute illness would have been excluded. Further, we do not have enough data to confirm or exclude CKDu in children with lower eGFR values. In addition, it is generally recommended to look for proteinuria rather than UACR for children as the later may underestimate proteinuria due to non-albumin proteins in the urine [16].

We recommend using our findings as baseline data only. Future studies should be looking in to proteinuria rather than albuminuria and require properly planned early morning samples. Further, a repeat sample is essential for confirmation of proteinuria. Use of new biomarkers to identify early renal insult among children in CKDu endemic area is recommended to investigate this issue.

## Conclusions

This study provides evidence to suggest that children in CKDu endemic regions are having an early renal damage. This observation needs to be investigated further in order to understand the worldwide epidemic of CKDu.

## Abbreviations

CINAC: Chronic Interstitial Nephritis in Agricultural Communities
CKDu: Chronic Kidney Disease of Unknown Origin
eGFR: Estimated Glomerular Filtration Rate
NCP: North Central Province
NKF: National Kidney Foundation
WHO: World Health Organization
